# Initial Biliary Drainage in Unresectable Bismuth Type III Malignant Hilar Obstruction: Comparative Effectiveness of ERCP and PTBD According to Drainage Adequacy in a Retrospective Two-Center Study

**DOI:** 10.3390/jcm15114146

**Published:** 2026-05-27

**Authors:** Berk Basş, Ömer Küçükdemirci

**Affiliations:** 1 Department of Gastroenterology and Hepatology, School of Medicine, Section of Internal Medicine, Aydın Adnan Menderes University, 09100 Aydın, Türkiye; 2 Department of Gastroenterology and Hepatology, School of Medicine, Section of Internal Medicine, Ondokuz Mayıs University, 55100 Samsun, Türkiye; drkucukdemirci@yahoo.com

**Keywords:** malignant hilar obstruction, bismuth type III, ERCP, PTBD, biliary drainage, drainage adequacy, cholangiocarcinoma

## Abstract

**Background**: Optimal biliary drainage in unresectable malignant hilar obstruction remains challenging, particularly in Bismuth type III disease due to complex biliary anatomy. Emerging evidence suggests that the adequacy of biliary decompression may be more important than the drainage modality itself in determining clinical outcomes. **Aim**: To compare the effectiveness of endoscopic retrograde cholangiopancreatography (ERCP) and percutaneous transhepatic biliary drainage (PTBD) in unresectable Bismuth type III malignant hilar obstruction, with particular emphasis on drainage adequacy. **Methods**: This retrospective two-center study included 199 patients with unresectable Bismuth type III malignant hilar obstruction (ERCP: *n* = 102; PTBD: *n* = 97). Drainage adequacy was defined as decompression of at least 50% of the non-atrophic liver using a segment-based anatomical approach. Bilirubin response was evaluated at predefined time points (days 7, 14, and 28). The primary outcome was biochemical response, while secondary outcomes included reintervention, complications, hospital stay, receipt of systemic therapy, and mortality. **Results**: Baseline characteristics were comparable between groups (mean age 66.8 ± 11.2 vs. 68.4 ± 10.7 years, *p* = 0.412; male sex 58.3% vs. 61.5%, *p* = 0.721). PTBD achieved significantly higher rates of adequate drainage than ERCP (*p* = 0.006). Although biochemical response rates were numerically higher in the PTBD group, multivariable analysis identified drainage adequacy—rather than drainage modality—as the strongest independent predictor of treatment success. Reintervention rates were significantly higher and time to reintervention significantly shorter in the ERCP group (*p* < 0.001). Cholangitis and post-procedural pain scores were more frequent following PTBD, whereas post-ERCP pancreatitis occurred exclusively after ERCP. No significant differences were observed in 30-day or 1-year mortality between groups. **Conclusions**: In unresectable Bismuth type III malignant hilar obstruction, drainage adequacy appears to be the principal determinant of clinical success. Although PTBD more frequently achieves adequate biliary decompression, outcomes seem to depend primarily on the effectiveness of drainage rather than the drainage modality itself.

## 1. Introduction

Perihilar cholangiocarcinoma (pCCA), also known as a Klatskin tumor, represents one of the most complex forms of biliary malignancy due to its anatomical location at the hepatic duct confluence and its tendency for longitudinal spread along the biliary tree. Patients frequently present at an advanced stage with obstructive jaundice, cholangitis, and limited resectability. Despite advances in surgical techniques, only a minority of patients—typically less than 20%—are eligible for curative resection at the time of diagnosis, making palliative biliary drainage a cornerstone of management in unresectable cases [1,2,3,4,5].

The anatomical classification of hilar biliary obstruction using the Bismuth–Corlette system remains clinically relevant, as it directly guides drainage strategies and procedural planning. Among these subtypes, Bismuth type III lesions pose a particular challenge due to involvement of either the right (type IIIa) or left (type IIIb) hepatic ductal systems, often requiring selective drainage of multiple liver segments. In such cases, the complexity of biliary anatomy significantly influences both the technical success of interventions and the likelihood of achieving effective biliary decompression [6,7,8].

Endoscopic retrograde cholangiopancreatography (ERCP) and percutaneous transhepatic biliary drainage (PTBD) are the two primary modalities used for palliative biliary drainage in malignant hilar obstruction. ERCP is widely utilized due to its minimally invasive nature and ability to provide internal drainage without external catheters, thereby improving patient comfort and quality of life. However, ERCP is associated with risks such as post-procedural pancreatitis and the potential for incomplete drainage, particularly in complex hilar strictures. In contrast, PTBD offers a higher technical success rate in certain anatomical scenarios and allows for targeted segmental drainage, although it may be associated with catheter-related complications and longer hospitalization in some patient populations [9,10,11,12].

A critical concept that has gained increasing recognition in recent years is that the success of biliary drainage should not be defined solely by the number or configuration of stents, but rather by the proportion of functional liver volume that is effectively drained. Several studies have demonstrated that drainage of at least 50% of the non-atrophic liver is associated with improved biochemical response, reduced incidence of cholangitis, and better overall clinical outcomes. Conversely, inadequate drainage may lead to persistent hyperbilirubinemia, increased risk of infection, and higher rates of reintervention [8,13,14,15].

Despite these advances, significant heterogeneity persists in the literature regarding the comparative effectiveness of ERCP and PTBD. Many previous studies have evaluated outcomes based on procedural characteristics—such as unilateral versus bilateral stenting or stent type—without adequately accounting for the actual volume of liver drained. This limitation is particularly relevant in Bismuth type III disease, where similar technical approaches may result in substantially different functional drainage outcomes depending on individual anatomy. As a result, comparisons between ERCP and PTBD may be confounded if drainage adequacy is not incorporated into the analysis [16,17,18].

Furthermore, inconsistencies in the definitions of clinical success, variability in outcome assessment timing, and differences in reporting complications such as cholangitis and pancreatitis further complicate interpretation of existing data. Standardized evaluation of biochemical response, reintervention rates, and complication profiles—particularly within anatomically homogeneous subgroups—is therefore essential to better understand the true comparative effectiveness of these drainage modalities [8,19]; studies focusing specifically on unresectable Bismuth type III malignant hilar obstruction are of particular importance. By limiting heterogeneity and incorporating drainage adequacy into outcome assessment, such analyses can provide more clinically meaningful insights into the optimal initial drainage strategy.

Therefore, the aim of the present study was to compare the effectiveness of ERCP and PTBD as initial biliary drainage modalities in patients with unresectable Bismuth type III malignant hilar obstruction, with a specific focus on the impact of achieving adequate drainage (defined as ≥50% of the non-atrophic liver) on biochemical response, reintervention rates, and clinical outcomes.

## 2. Methods

### 2.1. Study Design and Patient Population

This retrospective, two-center comparative study was conducted to evaluate the effectiveness of endoscopic and percutaneous biliary drainage in patients with unresectable Bismuth type III malignant hilar obstruction.

Consecutive patients who underwent initial biliary drainage using either endoscopic retrograde cholangiopancreatography (ERCP) or percutaneous transhepatic biliary drainage (PTBD) between January 2019 and December 2024 were screened. Patients were eligible if they had malignant hilar biliary obstruction classified as Bismuth type IIIa or IIIb and were deemed unresectable at the time of diagnosis. Unresectability was determined by a multidisciplinary hepatobiliary tumor board based on vascular invasion, bilateral ductal involvement, distant metastasis, inadequate future liver remnant, or poor performance status.

1. Inclusion and Exclusion Criteria

Inclusion criteria were: (1) age ≥ 18 years, (2) radiologically and histologically confirmed malignant hilar obstruction, (3) Bismuth type IIIa or IIIb classification, and (4) initial biliary drainage performed with ERCP or PTBD.

Patients were excluded if they had: (1) resectable disease at diagnosis (*n* = 56), (2) absence of histopathological confirmation (*n* = 48), (3) insufficient follow-up data for outcome assessment (*n* = 23), or (4) prior biliary intervention, chemotherapy, or surgical treatment before the index drainage procedure. Therefore, all included patients were treatment-naïve at the time of initial biliary drainage. After applying these criteria, a total of 199 patients were included in the final analysis.

2. Diagnostic Confirmation and Pre-Procedural Evaluation

All included patients had histologically confirmed cholangiocarcinoma. Tissue diagnosis was obtained via brush cytology and/or biopsy during ERCP or PTBD. In selected patients, additional sampling from lymph nodes, liver, or peritoneal lesions was performed when feasible.

Pre-procedural imaging using contrast-enhanced computed tomography and/or magnetic resonance cholangiopancreatography was reviewed to assess biliary anatomy, extent of ductal involvement, and the presence of atrophic liver segments. These findings were used to guide the drainage strategy, including selection of target ducts and avoidance of non-functional or atrophic segments.

3. Drainage Procedures

- ERCP Technique

ERCP procedures were performed by experienced endoscopists under conscious sedation or general anesthesia according to patient condition and institutional practice. Selective biliary cannulation was achieved using standard techniques.

The target ducts for drainage were determined prior to the procedure based on imaging findings, with the aim of maximizing drainage of functional liver parenchyma. Contrast injection into ducts that were not planned for drainage was avoided whenever possible.

The choice of stent type, including plastic or self-expandable metal stents, was made based on clinical and anatomical considerations, including expected survival, technical feasibility, and biliary anatomy. Unilateral or bilateral stenting was performed depending on the extent of ductal involvement and the feasibility of achieving adequate drainage.

Adjunctive techniques, including balloon dilatation and guidewire manipulation, were used when necessary to facilitate successful stent placement.

- PTBD Technique

PTBD procedures were performed under ultrasound and fluoroscopic guidance by experienced interventional radiologists. Access to the biliary system was obtained through the appropriate hepatic duct based on pre-procedural imaging.

Internal, external, or internal–external drainage catheters were placed according to anatomical and clinical considerations. The selection of drainage type was based on the extent of obstruction, patient condition, and technical feasibility. In selected cases, stents were placed during the same session or in a staged manner.

4. Drainage Strategy and Cross-Over

The initial drainage modality (ERCP or PTBD) was selected based on clinical assessment, anatomical considerations, and institutional practice. In the majority of patients, ERCP was performed as the first-line drainage approach. Direct PTBD was performed in a limited number of patients (*n* = 7) based on multidisciplinary tumor board decision, primarily in cases where anatomical complexity or anticipated technical difficulty suggested a higher likelihood of successful drainage via a percutaneous approach.

During ERCP, patients in whom biliary cannulation could not be achieved during the initial session underwent repeat ERCP attempts. Biliary cannulation was ultimately achieved in these patients; however, in a subset, the guidewire could not be advanced across the stricture, and these patients were referred for PTBD. In addition, patients who underwent technically successful ERCP with stricture traversal but failed to demonstrate a decrease in bilirubin levels during early follow-up were also referred for PTBD. Furthermore, patients who achieved partial biochemical improvement but did not reach complete response—particularly those considered eligible for systemic chemotherapy—underwent additional PTBD to optimize biliary drainage and facilitate oncologic treatment.

Therefore, cross-over from ERCP to PTBD was performed based on predefined technical and clinical criteria, including failure to traverse the stricture or inadequate biochemical response.

5. Drainage Adequacy

In all patients, the procedural goal was to achieve drainage of ≥50% of the functional, non-atrophic liver volume when technically and anatomically feasible. In patients who required cross-over from ERCP to PTBD, drainage adequacy was evaluated based on the final achieved drainage following all interventions.

Drainage adequacy was defined as decompression of ≥50% of the non-atrophic liver. Pre-procedural CT and/or MRCP findings were reviewed to evaluate biliary anatomy, segmental ductal involvement, and the presence of atrophic liver segments. The proportion of drained liver was estimated using a segment-based anatomical approach according to the Couinaud classification, integrating radiological reports, procedural findings, and post-procedural drainage distribution. Segments considered atrophic or non-functional on imaging were excluded from the estimation of functional liver volume.

Given the retrospective design, dedicated CT/MR volumetric software, manual volumetric segmentation, and interobserver agreement analysis were not performed. Therefore, drainage adequacy was assessed using a radiology-guided segmental approach rather than formal volumetric measurement, which may introduce some degree of estimation variability.

Patients were categorized into two groups according to drainage adequacy:

- Adequate drainage (≥50%);
- Inadequate drainage (<50%).

This variable was used as a key determinant in the analysis of clinical outcomes.

### 2.2. Outcome Definitions

#### 2.2.1. Primary Outcome

The primary outcome was biochemical response.

Baseline bilirubin was defined as the value measured immediately prior to the initial drainage procedure. Bilirubin levels were monitored during follow-up and assessed at predefined time points of 7, 14, and 28 days after the procedure. Early treatment failure was defined as no decrease or an increase in total bilirubin levels within the first 7 days after the initial drainage procedure. Partial response was defined as a reduction of at least 50% in total bilirubin compared to baseline values at 14 days. Complete response was defined as a total bilirubin level below 2 mg/dL at 28 days.

#### 2.2.2. Secondary Outcomes

Secondary outcomes included reintervention, time to reintervention, complications, length of hospital stay, receipt of oncologic treatment, and mortality.

Reintervention was defined as any additional endoscopic or percutaneous procedure performed due to inadequate drainage, stent dysfunction, or recurrent biliary obstruction. Time to reintervention was defined as the interval between the initial procedure and the first additional intervention.

Complications included cholangitis, pancreatitis, bleeding, and perforation. Cholangitis was defined according to the Tokyo Guidelines, and post-ERCP pancreatitis was defined according to the revised Atlanta classification.

Length of hospital stay was defined as the duration of hospitalization following the initial drainage procedure. In patients who required cross-over from ERCP to PTBD, hospitalization duration was calculated from the day of PTBD to ensure accurate comparison between groups, and days spent after the initial ERCP procedure were excluded from this analysis.

Receipt of oncologic treatment was defined as the initiation of systemic chemotherapy following biliary drainage.

Mortality was evaluated as both 30-day and 1-year mortality.

### 2.3. Statistical Analysis

Continuous variables were expressed as mean ± standard deviation or median with interquartile range, depending on distribution. Categorical variables were presented as frequencies and percentages.

Normality of distribution was assessed using appropriate statistical tests. Comparisons between groups were performed using independent samples tests for continuous variables and chi-square or Fisher’s exact tests for categorical variables.

Multivariable logistic regression analysis was performed to identify independent predictors of biochemical response. Variables included in the model were age, sex, baseline bilirubin level, drainage modality, and drainage adequacy.

A *p*-value of less than 0.05 was considered statistically significant. Statistical analyses were performed using IBM SPSS Statistics for Windows, version 29.0 (IBM Corp., Armonk, NY, USA).

#### Ethical Considerations

The study protocol was approved by the institutional ethics committee (Approval No.: 2025/333, Date: 16 December 2025), and the study was conducted in accordance with the principles of the Declaration of Helsinki. Written informed consent for the procedures and use of anonymized clinical data for research purposes was obtained from all patients.

Use of Generative Artificial Intelligence (GenAI): ChatGPT (OpenAI, San Francisco, CA, USA) was used solely for language editing, grammar correction, and improvement of manuscript readability during manuscript preparation. The AI tool was not used for data collection, statistical analysis, interpretation of results, or generation of scientific conclusions. All scientific content and final manuscript revisions were reviewed and approved by the authors.

## 3. Results

A total of 326 patients with suspected malignant hilar biliary obstruction were initially screened. Of these, 56 patients were excluded due to resectable disease, 48 due to lack of histopathological confirmation, and 23 due to insufficient follow-up data. Additionally, patients who had undergone prior biliary intervention, chemotherapy, or surgical treatment were excluded to ensure a treatment-naïve cohort.

After applying the inclusion and exclusion criteria, a total of 199 patients were included in the final analysis, of whom 102 underwent ERCP and 97 underwent PTBD as the initial drainage modality.

Among the patients in the PTBD group, 55 underwent PTBD due to inability to traverse the stricture during ERCP, 24 due to lack of bilirubin reduction despite successful stent placement, 11 due to partial biochemical response with a target of achieving complete response, and 7 were referred directly for PTBD based on multidisciplinary tumor board decision, as they were considered more likely to benefit from a percutaneous approach.

The baseline characteristics of patients were largely comparable between the ERCP and PTBD groups, indicating an overall balanced cohort suitable for comparative analysis. Age distribution and gender proportions did not differ significantly, suggesting that demographic factors were unlikely to confound outcome comparisons. Similarly, the distribution of Bismuth subtypes (IIIa vs. IIIb) was homogeneous across groups, ensuring anatomical comparability.

However, baseline bilirubin levels were significantly higher in the PTBD group, indicating that patients undergoing PTBD presented with more advanced biliary obstruction. This finding is clinically relevant, as higher bilirubin levels are often associated with more severe cholestasis and may influence both procedural selection and subsequent outcomes. Therefore, this variable was considered in further multivariable analyses to account for potential confounding effects (Table 1).

The reasons for unresectability are summarized in Table 2. Multiple factors were present in approximately 20% of patients. Key determinants such as vascular invasion, metastatic disease, and performance status were similarly distributed between groups, indicating comparable oncological burden (Table 2).

Procedural characteristics differed significantly between the ERCP and PTBD groups, reflecting inherent differences in technique and clinical application. ERCP procedures predominantly involved internal drainage, whereas PTBD demonstrated a heterogeneous distribution of internal, external, and combined drainage approaches. This variation highlights the flexibility of percutaneous techniques in addressing complex biliary anatomy.

The use of metal stents was significantly higher in the PTBD group, which may partly explain differences in long-term patency and reintervention rates observed later in the analysis. Although bilateral drainage was numerically more frequent in the PTBD group, this difference did not reach statistical significance. The crossover rate was higher in the ERCP group, suggesting a higher rate of initial technical or clinical failure requiring alternative intervention (Table 3).

This figure presents the comparative distribution of drainage adequacy between ERCP and PTBD groups, clearly demonstrating a higher proportion of patients achieving ≥50% drainage in the PTBD cohort. The visual separation between groups highlights the superior performance of PTBD in achieving effective decompression of functional liver volume.

The marked difference in adequate drainage rates supports the central hypothesis of the study, suggesting that the technical approach alone does not determine success, but rather the ability to achieve sufficient drainage of non-atrophic liver segments. The higher proportion of inadequate drainage observed in the ERCP group may reflect the limitations of endoscopic access in complex hilar anatomy, particularly in cases requiring multi-segmental drainage.

**Figure 1:**
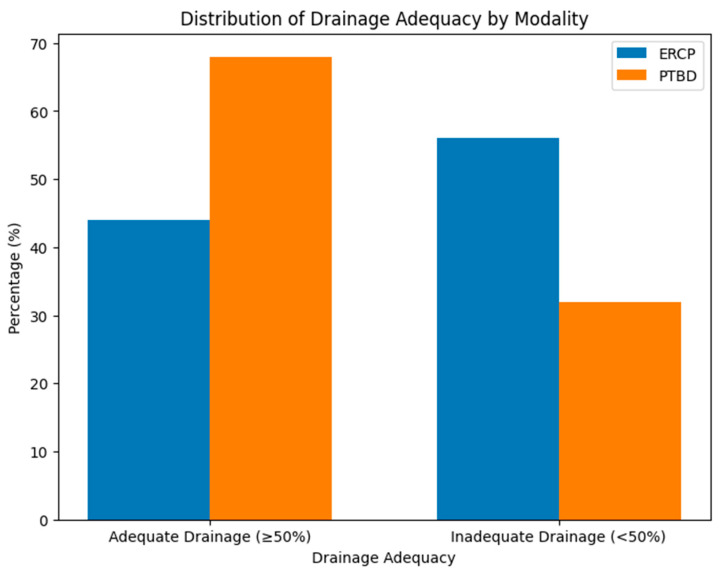
Distribution of Drainage Adequacy According to Drainage Modality.

From a clinical standpoint, this figure reinforces the importance of pre-procedural planning and imaging-based targeting of biliary segments. Achieving ≥50% drainage appears to be a critical threshold for therapeutic success, and this visual representation strengthens the argument that drainage adequacy should be prioritized over procedural modality when selecting the optimal intervention strategy (Figure 1).

A significantly higher proportion of patients in the PTBD group achieved adequate drainage compared to those in the ERCP group. This finding supports the hypothesis that percutaneous approaches may be more effective in achieving sufficient decompression of functional liver parenchyma, particularly in complex hilar obstructions.

The lower rate of adequate drainage in the ERCP group suggests limitations in selectively targeting multiple hepatic segments, especially in anatomically challenging cases. This discrepancy underscores the importance of evaluating drainage success not merely based on procedural execution, but on the actual physiological impact of the intervention, as reflected by effective liver decompression (Table 4).

Biochemical response was significantly better in the PTBD group compared to the ERCP group, with higher rates of both complete and partial response. These findings suggest that PTBD may provide more effective biliary decompression, particularly in patients with more advanced disease, as reflected by higher baseline bilirubin levels.

When analyzed according to drainage adequacy, the impact of achieving ≥50% drainage became even more pronounced. Complete and partial biochemical response rates were markedly lower in patients with inadequate drainage (<50%), regardless of drainage modality. In contrast, patients who achieved adequate drainage demonstrated substantially higher response rates. This finding highlights that drainage adequacy is a stronger determinant of biochemical success than the procedural approach itself, supporting the concept that effective liver decompression should be the primary goal of intervention (Table 5).

This finding suggests that PTBD provides more durable biliary decompression, likely due to its ability to achieve more effective drainage of multiple hepatic segments. In contrast, the steeper decline observed in the ERCP group indicates a higher likelihood of early stent dysfunction or inadequate initial drainage, necessitating repeat procedures.

**Figure 2:**
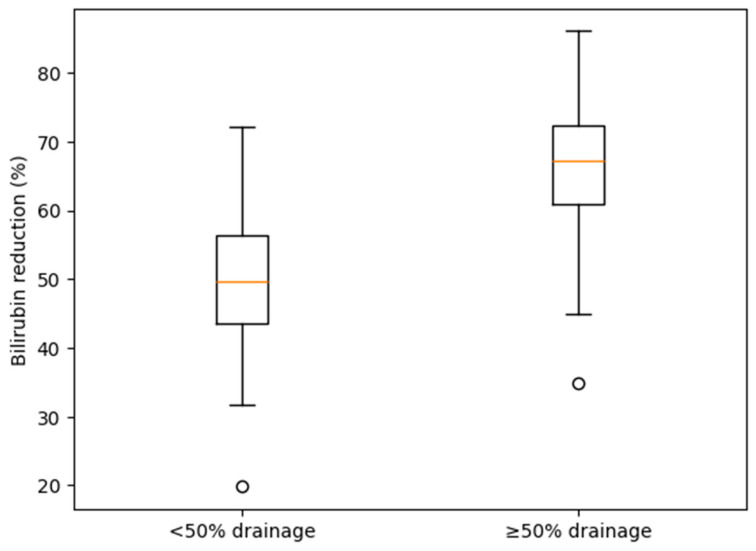
Biochemical Response According to Drainage Adequacy. Note: Adequate drainage was defined as a ≥50% reduction in serum bilirubin levels. In the boxplot, the orange horizontal line represents the median, the boxes indicate the interquartile range (IQR), whiskers represent the non-outlier range, and black circles denote outlier values.

From a clinical perspective, prolonged reintervention-free survival is highly relevant, as it reduces patient burden, minimizes procedural risks, and decreases healthcare utilization. These results reinforce the importance of selecting a drainage strategy that not only achieves initial success but also maintains long-term patency (Figure 2).

Reintervention rates were significantly higher in the ERCP group compared to the PTBD group, indicating a greater likelihood of recurrent biliary obstruction or insufficient initial drainage following endoscopic intervention. This finding is consistent with the observed lower rate of adequate drainage in the ERCP cohort and suggests that incomplete decompression may contribute to early failure.

Time to reintervention was also significantly shorter in the ERCP group, further supporting the notion that ERCP may be associated with reduced long-term patency in complex hilar obstructions. In contrast, the longer interval observed in the PTBD group reflects more durable drainage, likely attributable to more effective segmental decompression and higher utilization of metal stents (Table 6).

Complications were categorized into clinically meaningful groups according to drainage modality and severity (Table 7). Cholangitis was observed in both groups; however, severe cholangitis was more frequent in the PTBD group. Cholangitis severity was graded according to the Tokyo Guidelines, while post-procedural pancreatitis severity was assessed using the revised Atlanta classification.

Post-procedural pancreatitis occurred exclusively following ERCP, reflecting the inherent risks associated with endoscopic papillary manipulation. In contrast, PTBD-related complications included hemobilia, bile leak, catheter/stent dislodgement, and sepsis. In addition, post-procedural VAS pain scores were significantly higher in the PTBD group, indicating a greater burden of external drainage-related discomfort.

Although overall complication rates were higher in the PTBD group, the complication profiles differed substantially between modalities, highlighting the trade-off between infectious, catheter-related, and procedure-specific adverse events.

No statistically significant difference was observed between the ERCP and PTBD groups in terms of short-term or long-term mortality. This finding suggests that, despite differences in drainage success and complication profiles, overall survival may be more strongly influenced by disease biology and oncological factors rather than the drainage modality itself.

The clinical relevance of biliary drainage should also be interpreted within a broader oncological context. During follow-up, systemic therapy was administered in 88 patients (44.2%). The proportion of patients receiving chemotherapy after drainage was 31.4% in the ERCP group and 57.7% in the PTBD group. These findings highlight the potential role of effective biliary decompression in facilitating subsequent oncological treatment. However, the length of hospital stay was significantly longer in the PTBD group, likely reflecting the need for catheter management, staged procedures, and monitoring for procedure-related complications. This difference has important clinical implications, particularly in terms of healthcare resource utilization and patient quality of life (Table 8).

This figure highlights the critical impact of drainage adequacy on biochemical outcomes. Patients who achieved drainage of at least 50% of the non-atrophic liver demonstrated significantly greater reductions in bilirubin levels compared to those with inadequate drainage.

The clear separation between groups emphasizes that effective decompression of functional liver volume is the primary determinant of biochemical success. While procedural modality plays a role, the extent of drainage appears to be a more decisive factor in achieving clinically meaningful improvement.

These findings provide strong visual support for the study’s central hypothesis and align with the results of multivariable analysis, in which drainage adequacy emerged as the strongest independent predictor of treatment success. Clinically, this underscores the need to prioritize strategic planning of drainage procedures to maximize liver volume decompression rather than focusing solely on technical approach (Figure 2).

Multivariable analysis demonstrated that achieving adequate drainage was the strongest independent predictor of biochemical response. Patients with drainage of at least 50% of the non-atrophic liver were nearly four times more likely to achieve a successful biochemical outcome compared to those with inadequate drainage. This finding remained robust after adjustment for potential confounders.

In contrast, the drainage modality itself was not an independent predictor of biochemical success after adjustment, suggesting that the effectiveness of biliary decompression is primarily determined by the extent of functional liver drainage rather than the procedural approach. These results provide strong evidence supporting a shift in clinical focus from technique selection to optimization of drainage adequacy (Table 9).

## 4. Discussion

In this two-center comparative analysis of patients with unresectable Bismuth type III malignant hilar obstruction, several important findings emerged. First, PTBD was associated with significantly higher rates of adequate drainage compared to ERCP. Second, biochemical response rates were superior in the PTBD group; however, when adjusted for confounding factors, drainage adequacy—rather than the drainage modality itself—was identified as the strongest independent predictor of treatment success. Third, ERCP was associated with higher reintervention rates and shorter time to reintervention, whereas PTBD demonstrated longer drainage durability but at the expense of higher cholangitis rates and prolonged hospitalization.

One of the most clinically relevant findings of this study is the central role of drainage adequacy. Achieving decompression of at least 50% of the non-atrophic liver significantly increased the likelihood of both complete and partial biochemical response. This observation is consistent with previous studies demonstrating that the extent of functional liver drainage is a critical determinant of clinical success in malignant hilar obstruction In particular, Takahashi and colleagues emphasized that inadequate drainage is strongly associated with persistent hyperbilirubinemia and increased complication rates, while Xia et al. highlighted the prognostic importance of effective biliary decompression in improving patient outcomes [14,15]. In the present study, drainage adequacy was assessed using a segment-based anatomical approach rather than formal volumetric analysis. While this method reflects real-world clinical decision-making and integrates both imaging and procedural findings, it may be associated with some degree of estimation variability. In addition, we observed that in a subset of patients within the ERCP group, adequate drainage (≥50%) did not consistently translate into the expected biochemical response. In some of these cases, subsequent PTBD was associated with improved bilirubin reduction. This observation may indicate that, beyond the extent of drainage alone, factors related to the functionality and effectiveness of the selected drainage approach could also play a role in clinical outcomes. Similar observations have been reported previously, suggesting that technical success alone may not always translate into effective functional biliary decompression, particularly in complex hilar strictures where drainage distribution and liver functionality are highly heterogeneous [13,19].

Importantly, our multivariable analysis demonstrated that drainage modality was not an independent predictor of biochemical response after adjustment for drainage adequacy. This finding supports the growing body of literature suggesting that the success of biliary drainage should be evaluated based on functional outcomes rather than procedural characteristics alone [16,18]. In complex hilar strictures such as Bismuth type III lesions, the same technical approach may result in markedly different clinical outcomes depending on how effectively the functional liver volume is drained. Therefore, comparisons between ERCP and PTBD that do not account for drainage adequacy may be inherently biased.

The higher rate of adequate drainage observed in the PTBD group in this study is likely attributable to the technical advantages of percutaneous access. PTBD allows for more precise segmental targeting based on pre-procedural imaging and facilitates drainage of multiple hepatic sectors, particularly in anatomically complex strictures. This is in line with previous reports indicating higher technical success rates for PTBD in advanced hilar obstruction [9,10]. In addition, primary percutaneous stenting above the ampulla has been proposed as a potential alternative strategy in selected anatomically complex hilar strictures, potentially avoiding transpapillary manipulation and reducing pancreatitis risk [20]. However, the optimal drainage approach should remain individualized according to biliary anatomy and drainage feasibility. In contrast, ERCP may be limited by difficulty in selective cannulation of multiple ducts and the risk of contrast injection into undrained segments, which can predispose to cholangitis. In contrast, ERCP may be limited by difficulty in selective cannulation of multiple ducts and the risk of contrast injection into undrained segments, which can predispose to cholangitis.

Reintervention outcomes further support these findings. Patients in the ERCP group required significantly more frequent repeat procedures and experienced shorter intervals to reintervention. This suggests that, in addition to lower initial drainage adequacy, ERCP may be associated with reduced long-term patency in this patient population. Previous studies have similarly reported higher reintervention rates following endoscopic drainage, particularly when plastic stents are used or when drainage is incomplete [17,19].

The complication profiles observed in this study highlight the trade-offs between the two modalities. Although certain complications, such as cholangitis and bleeding, may occur following both ERCP and PTBD, the frequency and severity of these events can differ substantially depending on the drainage technique, extent of biliary decompression, and procedural characteristics [21,22,23]. PTBD was associated with a higher incidence of cholangitis, which may be explained by catheter-related factors, including external drainage, repeated manipulation, and potential bacterial colonization. In addition, PTBD-specific complications such as hemobilia, catheter-related discomfort, bile leak, and sepsis were observed more frequently in the percutaneous group. This finding contrasts with some reports suggesting higher cholangitis rates following ERCP, underscoring the complexity of this issue and the influence of procedural technique, drainage completeness, and patient selection [18]. Conversely, post-procedural pancreatitis was observed exclusively in the ERCP group, reflecting the inherent risks associated with endoscopic papillary intervention. The higher post-procedural VAS pain scores observed in the PTBD group further emphasize the clinical burden associated with external drainage catheters and should be considered when selecting the optimal drainage strategy. Overall, these findings suggest that different drainage modalities are associated with distinct complication patterns, and that the choice of intervention should consider not only technical success and drainage adequacy, but also the expected profile of procedure-related adverse events.

Despite these differences, no significant differences were observed in short-term or long-term mortality between the two groups. However, these findings should be interpreted with caution, as survival outcomes in patients with unresectable malignant hilar obstruction are strongly influenced by multiple factors, including tumor biology, disease stage, and access to systemic therapy, which were not fully captured in this study. Similar findings have been reported in previous studies, where improvements in drainage success did not necessarily translate into survival benefit [5,7]. The clinical relevance of biliary drainage should be interpreted within the broader oncological context. Several contemporary studies evaluating malignant hilar obstruction have included patients diagnosed on the basis of clinical and radiological findings without histopathological confirmation, reflecting the inherent diagnostic complexity of hilar cholangiocarcinoma in real-world practice. Establishing tissue diagnosis in proximal biliary strictures remains challenging because of the submucosal growth pattern of the tumor, desmoplastic stromal reaction, and the relatively limited sensitivity of conventional brush cytology and forceps biopsy techniques [7,20]. At the same time, several benign biliary disorders—including IgG4-related sclerosing cholangitis, primary sclerosing cholangitis, and inflammatory hilar strictures—may closely mimic perihilar cholangiocarcinoma both radiologically and clinically. In the present study, we deliberately restricted inclusion to histopathologically confirmed malignancies in order to minimize diagnostic misclassification and preserve the internal validity of the comparative analysis. Although this strategy may have reduced external generalizability to certain real-world cohorts in which treatment decisions are frequently based on clinicoradiological assessment alone, it allowed for a more diagnostically homogeneous population and reduced the risk of confounding by benign biliary disease. Biliary decompression is not an endpoint in itself; rather, its value lies in relieving cholestasis, enabling systemic therapy when feasible, and providing symptom palliation in patients who are not candidates for oncological treatment. In the present cohort, no patient had received chemotherapy before drainage, whereas 44.2% received systemic therapy after biliary decompression. This finding highlights the role of effective drainage as a bridge to oncological treatment in selected patients. In addition, access to systemic therapy was more frequent in the PTBD group, which may reflect the higher rates of adequate drainage achieved with the percutaneous approach. More effective biliary decompression may facilitate earlier bilirubin reduction and improve eligibility for systemic treatment in selected patients [24]. Previous studies have emphasized that patients with unresectable perihilar cholangiocarcinoma require biliary drainage not only for symptom relief but also to allow palliative systemic chemotherapy [25]. In addition, chemotherapy after PTBD has been associated with improved overall survival in patients with malignant biliary obstruction, supporting the role of effective biliary decompression as a bridge to systemic therapy [26].

Another notable finding is the longer hospital stay observed in the PTBD group. This may reflect the need for catheter management, staged interventions, and monitoring for complications. While ERCP may offer advantages in terms of shorter hospitalization and patient comfort due to internal drainage, these benefits must be balanced against the higher likelihood of reintervention and potentially inadequate drainage in complex cases.

This study has several clinical implications. First, it supports a paradigm shift from technique-centered decision-making to outcome-centered planning, where the primary goal is achieving adequate drainage of functional liver volume. Second, it suggests that PTBD may be preferred in cases where achieving ≥50% drainage via ERCP is unlikely. Third, it highlights the importance of careful pre-procedural imaging and multidisciplinary planning to optimize drainage strategy.

Several limitations of this study should be acknowledged. The retrospective design introduces the potential for selection bias, and the choice of drainage modality was not randomized. In addition, mortality outcomes should be interpreted with caution due to the lack of comprehensive oncological and clinical data, including metastatic burden, systemic therapy characteristics, performance status, and cause of death. Baseline differences, including higher bilirubin levels in the PTBD group, may reflect more advanced disease and could have influenced outcomes despite statistical adjustment.

Additionally, the estimation of drainage adequacy was based on imaging and procedural findings rather than formal volumetric analysis, which may have introduced some degree of measurement variability. Furthermore, the observation that adequate drainage did not consistently result in biochemical response in all patients suggests that drainage adequacy alone may not fully capture functional biliary decompression.

Only patients with histopathologically confirmed malignancy were included in the study. Although this approach improved diagnostic certainty and minimized the risk of including benign biliary strictures or alternative pathologies, it may have reduced the generalizability of the findings to real-world cohorts in which clinical and radiological diagnosis is frequently used in the absence of tissue confirmation.

In patients who initially underwent ERCP and subsequently required PTBD, hospitalization days prior to PTBD were attributed to the ERCP group, while hospitalization duration for PTBD was recalculated beginning from the day of the percutaneous procedure. Nevertheless, despite this effort to minimize bias, hospitalization comparisons between groups may still have been influenced by the greater clinical complexity of patients ultimately requiring PTBD.

Finally, variations in operator experience and institutional protocols may also have influenced procedural outcomes.

In patients with unresectable Bismuth type III malignant hilar obstruction, drainage adequacy is the most important determinant of biochemical success. While PTBD is more likely to achieve adequate drainage and provide durable biliary decompression, the choice of modality should be individualized based on the likelihood of achieving effective drainage. These findings underscore the need for a strategic, anatomy-based approach to biliary drainage rather than reliance on procedural preference alone.

## 5. Conclusions

In patients with unresectable Bismuth type III malignant hilar obstruction, the adequacy of biliary drainage emerges as the most critical determinant of biochemical success. Achieving drainage of at least 50% of the non-atrophic liver significantly improves treatment response, regardless of the drainage modality used.

However, PTBD was more frequently associated with achieving adequate drainage. Although PTBD demonstrated higher rates of adequate drainage and longer reintervention-free intervals, it was associated with increased cholangitis rates and prolonged hospitalization. In contrast, ERCP offered advantages in terms of shorter hospital stay but was linked to higher reintervention rates and lower drainage adequacy in complex hilar anatomy.

These findings suggest that the choice of drainage modality should be guided primarily by the likelihood of achieving effective liver decompression rather than procedural preference alone. A strategy focused on maximizing functional liver drainage may lead to improved clinical outcomes in this challenging patient population.

## Figures and Tables

**Table 1:** Baseline Demographic and Clinical Characteristics.

Variable	ERCP (*n* = 102)	PTBD (*n* = 97)	*p*-Value
Age (years, mean ± SD)	66.8 ± 11.2	68.4 ± 10.7	0.412
Gender (Male, %)	59 (58.3%)	60 (61.5%)	0.721
Baseline bilirubin (mg/dL)	14.6 ± 5.2	16.1 ± 6.3	0.038
ASA score (≥3, %)	44 (42.7%)	47 (48.1%)	0.503
Bismuth type IIIa (%)	52 (51.2%)	46 (47.8%)	0.689
Bismuth type IIIb (%)	50 (48.8%)	51 (52.2%)	0.689

Abbreviations: ASA: American Society of Anesthesiologists; SD: standard deviation; ERCP: endoscopic retrograde cholangiopancreatography; PTBD: percutaneous transhepatic biliary drainage.

**Table 2:** Reasons for Unresectability According to Drainage Modality.

Reason for Unresectability	ERCP *n* (%)	PTBD *n* (%)
Multiple factors	20 (19.8%)	20 (20.4%)
N2 lymph node metastasis	12 (11.9%)	11 (11.2%)
FLR insufficiency	13 (12.9%)	12 (12.2%)
Poor performance status	16 (15.8%)	10 (10.2%)
Hepatic artery invasion	13 (12.9%)	7 (7.1%)
Portal vein involvement	3 (3.0%)	10 (10.2%)
Peritoneal carcinomatosis	6 (5.9%)	5 (5.1%)
Distant metastasis	6 (5.9%)	4 (4.1%)

**Table 3:** Procedural Characteristics and Drainage Strategy.

Variable	ERCP (*n* = 102)	PTBD (*n* = 97)	*p*-Value
Plastic stent (%)	64 (62.4%)	—	—
Metal stent (%)	38 (37.6%)	53 (54.2%)	0.041
Internal drainage (%)	102 (100%)	37 (38.5%)	<0.001
External drainage (%)	—	34 (34.6%)	—
Internal-external (%)	—	26 (26.9%)	—
Unilateral drainage (%)	73 (71.8%)	62 (63.5%)	0.298
Bilateral drainage (%)	29 (28.2%)	35 (36.5%)	0.298

**Table 4:** Drainage Adequacy According to Modality.

Variable	ERCP	PTBD	*p*-Value
≥50% drainage (%)	44.3	68.2	0.006
<50% drainage (%)	55.7	31.8	0.006

**Table 5:** Biochemical Response According to Drainage Modality and Adequacy.

Outcome	ERCP *n* (%)	PTBD *n* (%)	*p*-Value
Complete response	29 (28.6%)	40 (41.3%)	0.048
Partial response	47 (46.2%)	62 (63.5%)	0.021
No response	26 (25.2%)	14 (14.2%)	0.039
	≥50% drainage *n* (%)	<50% drainage *n* (%)	*p*-value
Complete response	72 (52.8%)	9 (14.8%)	<0.001
Partial response	97 (71.4%)	18 (29.5%)	<0.001

**Table 6:** Reintervention and Stent-Related Outcomes.

Variable	ERCP	PTBD	*p*-Value
Reintervention (%)	78.4	32.7	<0.001
Time to reintervention (days, mean ± SD)	92.6 ± 28.4	158.3 ± 35.7	<0.001
Stent occlusion (%)	64.2	28.8	<0.001
Stent migration (%)	11.3	6.7	0.284

**Table 7:** Complications According to Drainage Modality.

Complication	ERCP *n* (%)	PTBD *n* (%)	*p*-Value
Cholangitis–mild	24 (23.5%)	21 (21.6%)	
Cholangitis–severe	5 (4.9%)	14 (14.4%)	
Pancreatitis–mild	2 (2.0%)	0	
Pancreatitis–severe	1 (1.0%)	0	
Bleeding	5 (4.9%)	1 (1.0%)	
Perforation	3 (2.9%)	2 (2.1%)	
Hemobilia	0	4 (4.1%)	
Sepsis	5 (4.9%)	14 (14.4%)	
Catheter/Stent dislodgement	0	5 (5.2%)	
Bile leak	0	2 (2.1%)	
Post-procedural VAS pain score, mean ± SD	3.4 ± 1.6	6.8 ± 1.7	<0.001
Overall complication rate	35 (34.7%)	47 (48.1%)	0.048

**Table 8:** Mortality and Clinical Outcomes.

Outcome	ERCP (%)	PTBD (%)	*p*-Value
30-day mortality	14.2	16.3	0.684
1-year mortality	68.5	65.1	0.571
Length of hospital stay (days)	7.8 ± 3.2	11.6 ± 4.5	<0.001

**Table 9:** Multivariable Logistic Regression Analysis for Predictors of Biochemical Response.

Variable	Odds Ratio (OR)	95% CI	*p*-Value
Age	0.98	0.95–1.02	0.341
Gender (Male)	1.12	0.64–1.96	0.689
Baseline bilirubin	0.91	0.86–0.96	0.002
ERCP (vs. PTBD)	0.72	0.41–1.28	0.264
≥50% drainage	3.84	2.01–7.32	<0.001

## Data Availability

The data presented in this study are available from the corresponding author upon reasonable request.
